# Supplementary material for: Preparation of Ag Doped Keratin/PA6 Nanofiber Membrane with Enhanced Air Filtration and Antimicrobial Properties
Source: Polymers (Basel). 2019 Sep 16;11(9):1511. doi: 10.3390/polym11091511 (PMC6780504; doi:10.3390/polym11091511)
Supplement: Supplementary file 1 [file polymers-11-01511-s001.pdf]

Preparation of Ag Doped Keratin/PA6 Nanofiber Membrane with Enhanced Air Filtration and Antimicrobial Properties

Baolei Shen [1], Dongyu Zhang [2], Yujuan Wei [1], Zihua Zhao [3], Xiaofei Ma [1], Xiaodan Zhao [3], Shuo Wang [1,*] and Wenxiu Yang [1,*]

[1] College of Textile and Garments, Hebei University of Science and Technology, Shijiazhuang, 050018, China; shenbaolie@163.com. (B.S.); weiyujuan66@sina. com. (Y.W.); mxiaof1007@163.com. (X.M.)
[2] School of Environmental Science and Engineering, Hebei University of Science and Technology, Shijiazhuang, 050018, China; dongyuz01@163.com
[3] College of Bioscience and Bioengineering, Hebei University of Science and Technology, Shijiazhuang, 050018, China; zzh1398921@163.com. (Z.Z.); zxd199404@163.com. (X.Z.)
* Correspondence: fzwangshuo@hebust.edu.cn (S.W.); wenxiuyang-hbust@outlook.com (W.Y.); Tel.: +86-311-81668835 (S.W.); +86-311-81668836 (W.Y.)

Comparison of coarse wool and commercial wool

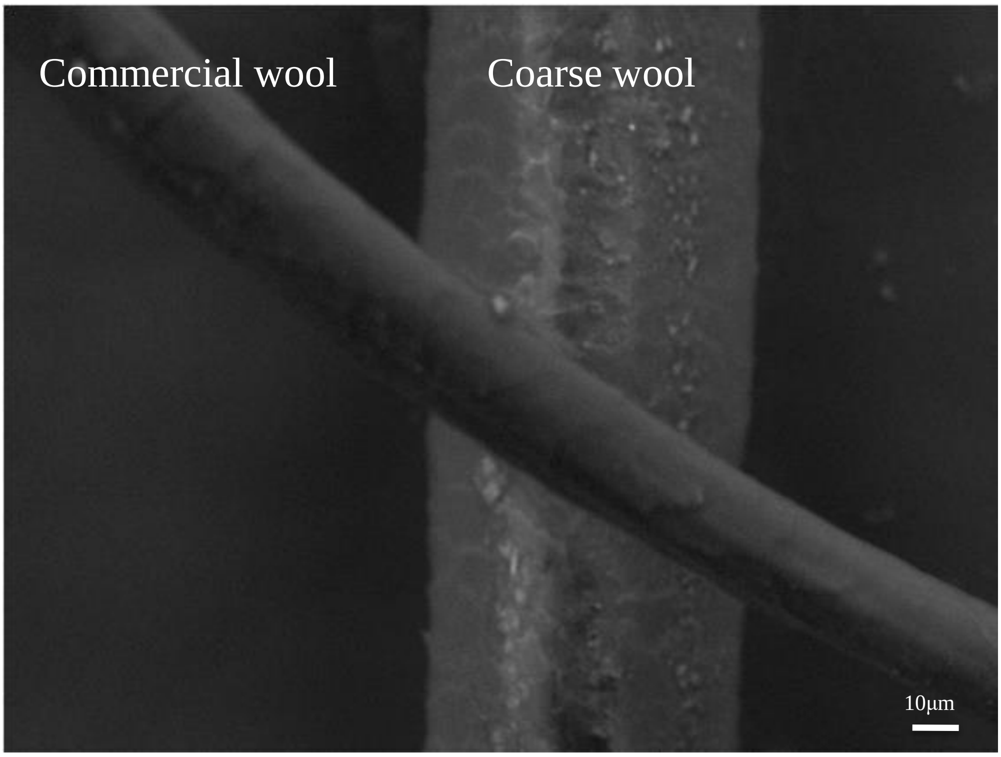

Figure S1. SEM images of coarse wool and commercial wool

SDS-PAGE

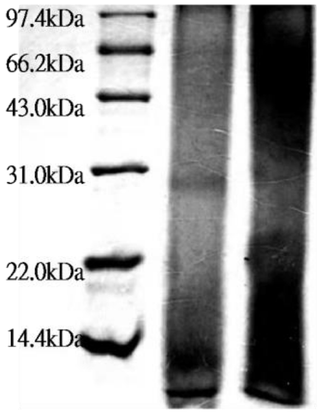

Figure S2. Schematic diagram of SDS-PAGE gel electrophoresis. The left is the low weight standard protein (marker), the middle is the method of $Na_2S_2O_3$ keratin electrophoresis image and the right is the method of $NaHSO_3$ keratin electrophoresis image.

Pore size distribution of the composite membrane

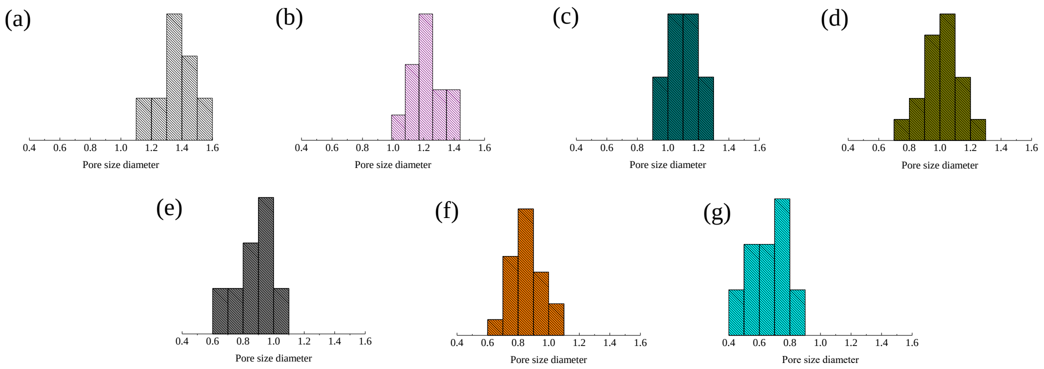

Figure S3. Schematic diagram of pore size distribution of composite membrane, The a, b, c, d, e f and g column images of Ker.0%/PA6 Ker.30%/PA6', Ker.30%/PA6 Ag.1%-Ker.30%/PA6, Ag.10%-Ker.30%/PA6, Ker.50%/PA6 and Ker.70%/PA6, respectively.

Filter efficiency and pressure drop

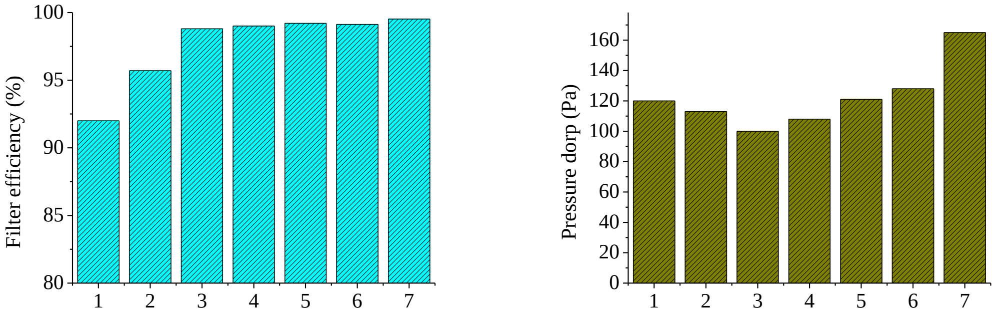

Figure S4. Schematic diagram of filter efficiency and pressure drop. The 1, 2, 3, 4, 5, 6 and 7 column images of Ker.0%/PA, Ker.30%/PA6', Ker.30%/PA6, Ag.1%-Ker.30%/PA6, Ag.10%-Ker.30/PA6, Ker.50%/PA6 and Ker.70%/PA6, respectively.

Table S1. Pore size diameter, WVT, and QF of nano-structure mats.

Samples	Pore size diameter (μm)	WVT (g/m2)	QF
Pure PA6	1.398±0.2	151.2	0.021
Ker.30%/PA6′	1.240±0.3	155.6	0.028
Ker.30%/PA6	1.113±0.2	166.2	0.044
Ag.1%-ker.30%/PA6	1.005±0.2	163.3	0.042
Ag.10%-ker.30%/PA6	0.8784±0.2	157.8	0.04
Ker.50%/PA6	0.8487±0.2	154.4	0.037
Ker.70%/PA6	0.6330±0.2	146.2	0.032

Thermal stability

Table S2. Mass of **residues**, peak degradation temperatures and melting temperatures of the different nanofibers mats.

Samples	Mass of residues (%)	Peak degradation temperatures (℃)
Pure PA6	0.92	434
Ker.30%/PA6′	2.04	436
Ker.30%/PA6	2.29	432
Ag.1%-ker.30%/PA6	22.6	417
Ag.10%-ker.30%/PA6	30.1	411
Ker.50%/PA6	8.21	426
Ker.70%/PA6	13.27	408

Table S3. The peak melting temperature of the composite membrane.

Samples	Peak melting temperature (℃)		
Pure PA6	207		218
Ker.30%/PA6′	214		225
Ker.30%/PA6	219		230
Ag.1%-ker.30%/PA6	215		230
Ag.10%-ker.30%/PA6		196	
Ker.50%/PA6	212		229
Ker.70%/PA6		209	
